# Supplementary material for: HIPP neurons in the dentate gyrus mediate the cholinergic modulation of background context memory salience
Source: Nat Commun. 2017 Aug 4;8:189. doi: 10.1038/s41467-017-00205-3 (PMC5543060; doi:10.1038/s41467-017-00205-3)
Supplement: Supplementary file 1 — Supplementary information [file 41467_2017_205_MOESM1_ESM.pdf]

File Name: Supplementary Information
Descriptions: Supplementary Figures and Supplementary Tables

File Name: Peer Review File
Descriptions:

Supplementary Information

Supplementary Figures

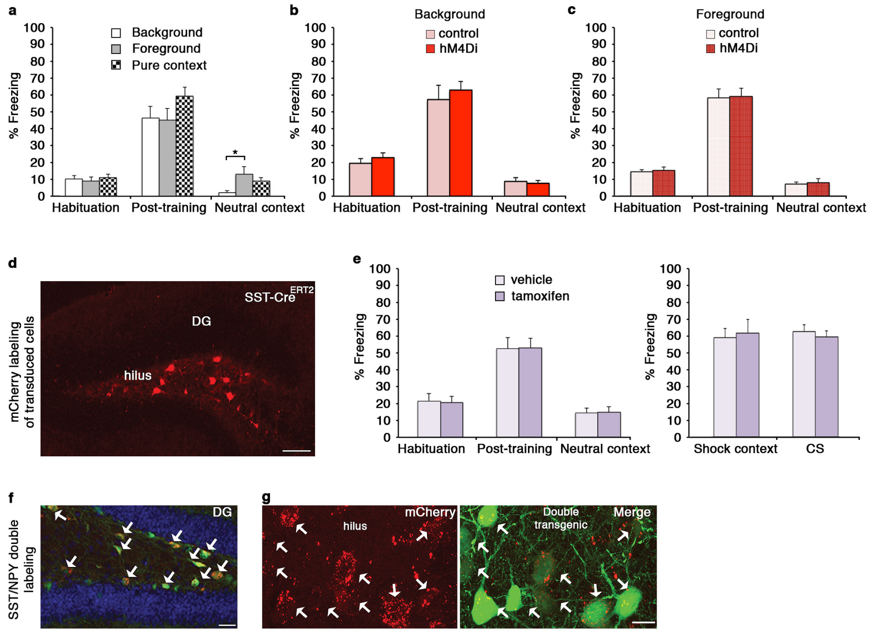

Supplementary Figure 1. Specificity of background context conditioning and HIPP cell manipulation (**a**) In C57Bl/6 mice no difference in freezing levels is observed during pre-training habituation and in the immediate post-training period between mice undergoing background context conditioning (*n*=8), foreground context conditioning (*n*=8) or pure context conditioning (*n*=8). In the neutral context, freezing levels after foreground conditioning are higher than after background conditioning, but both still remain at or below levels of the pre-training habituation phase. Further, the pharmacogenetic silencing of HIPP cells during (**b)** background context conditioning (control *n*=9; hM4Di *n*=8) or (**c**) foreground context conditioning (control *n*=7; hM4Di *n=8*) does not affect freezing behavior in the immediate post-training period, during habituation or in the neutral context. (**d**) A representative microscopic image shows viral expression of the mCherry tag in the hilus. Scale bar, 100 μm. We have carefully established the tamoxifen induction

protocol and indeed found that by contrast to the original publication[58], CRE recombination can be activated in virtually all SST positive cells of the injected area using our 3-day injection procedure. **e**) Tamoxifen injection to SST-Cre^{ERT2} mice without prior virus application (n=11) does not alter background context conditioning compared to controls receiving corresponding vehicle injections (n=11). (**f**) Immunohistochemical staining for SST in NPY-GFP mice. The quantification of double labeling reveals that 80.2 ± 2.4% of SST immunoreactive cells also express transgenic GFP as a marker of NPY gene expression (n=7, n=64 ± 2 cells per animal). Green, GFP; red, SST; blue, DAPI. Scale bar 20 µm. (**g**) Double-labeling of GFP and mCherry in NPY-GFP/SST-Cre^{ERT2} double transgenic mice with injection of AAVs to the hilus confirms a selective manipulation of the majority of GFP^{+} cells. Green, GFP; red, mCherry. Arrows indicate prominent double labeling. Scale bar, 10 µm. Data are means + s.e.m. Statistical analysis was done with Student's unpaired *t*-test. *$P < 0.05$.

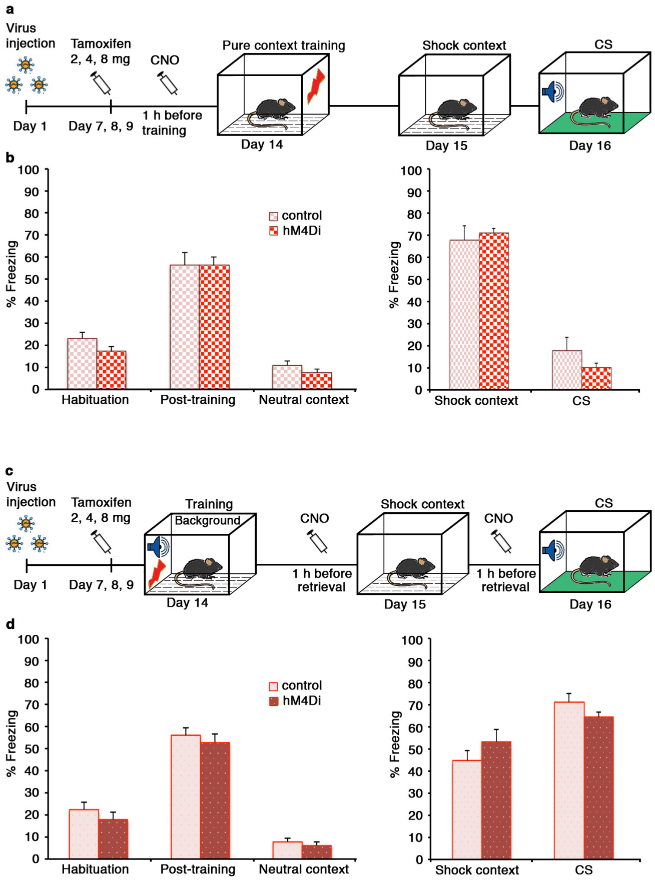

Supplementary Figure 2. HIPP cell inactivation does not affect pure context conditioning or context memory retrieval (**a**) A schematic of the pure context training paradigm. (**b**) (Left) No effect of CNO application concerning freezing behavior during the training phase or in the neutral context was observed in hM4Di transduced SST-CRE^{ERT2} driver mice (*n*=8) compared to animals injected with control vector (*n*=7). (Right) Both groups showed similar high levels of context memory and little response to the CS. (**d**) A schematic of the behavioral paradigm for testing potential effects of HIPP cell inactivation during memory retrieval. (Left) No effect of CNO application was observed in hM4Di mice (*n*=10) compared to controls (*n*=10) concerning freezing behavior in the training phase or in the neutral context. (Right) Neither background context memory nor auditory cued memory retrieval was affected in hM4Di transduced mice. Data are means + s.e.m. Statistical analysis was done with Student's unpaired *t*-test.

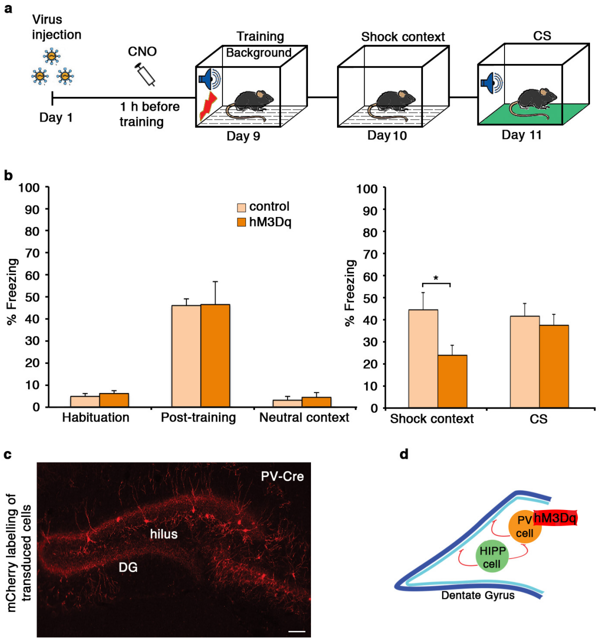

Supplementary Figure 3. Activation of parvalbumin interneurons in the DG also reduces background context memory (**a**) A schematic of the behavioral paradigm. (**b**) (Left) No effect of CNO application was observed in hM3Dq transduced PV-Cre mice (*n*=8) compared to mice with control virus (*n*=8) concerning freezing behavior during the training phase or in the neutral context. (Right) By contrast, activation of parvalbuminergic interneurons with CNO during training led to a reduction in background context memory without affecting auditory cued memory, contrasting the effect of HIPP cell inactivation. An inhibition of parvalbuminergic neurons thus cannot account for the observed effects of HIPP cell inactivation. However, inhibition of DG granule cells in general may attenuate background context memory formation. (**c**) A representative microscopic image shows expression of the virally expressed mCherry tag in hilar cell bodies and neuropil particularly in the DG granule cell layer. Scale bar, 100 μm. (**d**) A schematic of the proposed circuitry. Data are means + s.e.m. Statistical analysis was done with Student's unpaired *t*-test. $*P < 0.05$.

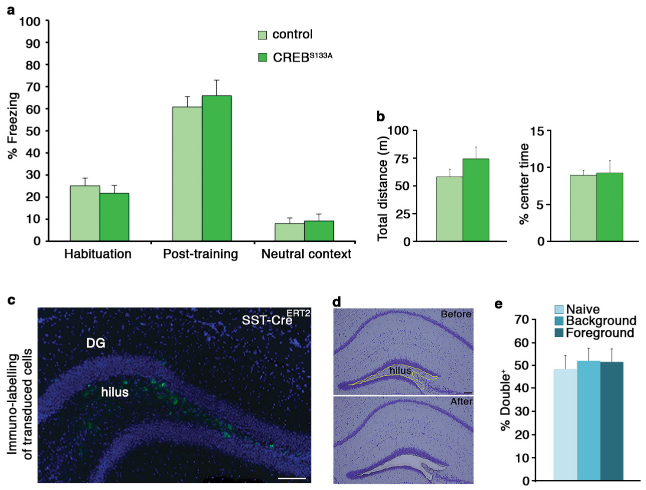

Supplementary Figure 4. Specificity of CREB^{S133A} effects (**a**) The viral transduction of HIPP cells with CREB^{S133A} does not affect freezing behavior in the immediate post-training period, during habituation or in the neutral context of SST-Cre^{ERT2} mice (**b**) (Left) Locomotor activity and (right) anxiety-like behavior tested in an open field are unchanged in mice with CREB^{S133A} expression in HIPP cells (n=8; controls n=10). (**c**) A representative microscopic image of the immunolabeling shows specific viral construct expression in the hilus. Scale bar, 100 μm. (**d**) Representative microscopic images show the dorsal hilus before and after tissue capture for quantitative mRNA expression analysis. Scale bar, 100 μm. (**e**) As opposed to the selective change in the hilus, the percentage of pCREB^{S133}/GFP double-positive cells in area CA1 is not altered 1 h after either background or foreground context conditioning compared to naïve controls (n=6 each). Data are means + s.e.m. Statistical analysis was done with Student's unpaired t-test (**a, b**) and one-way ANOVA (**e**).

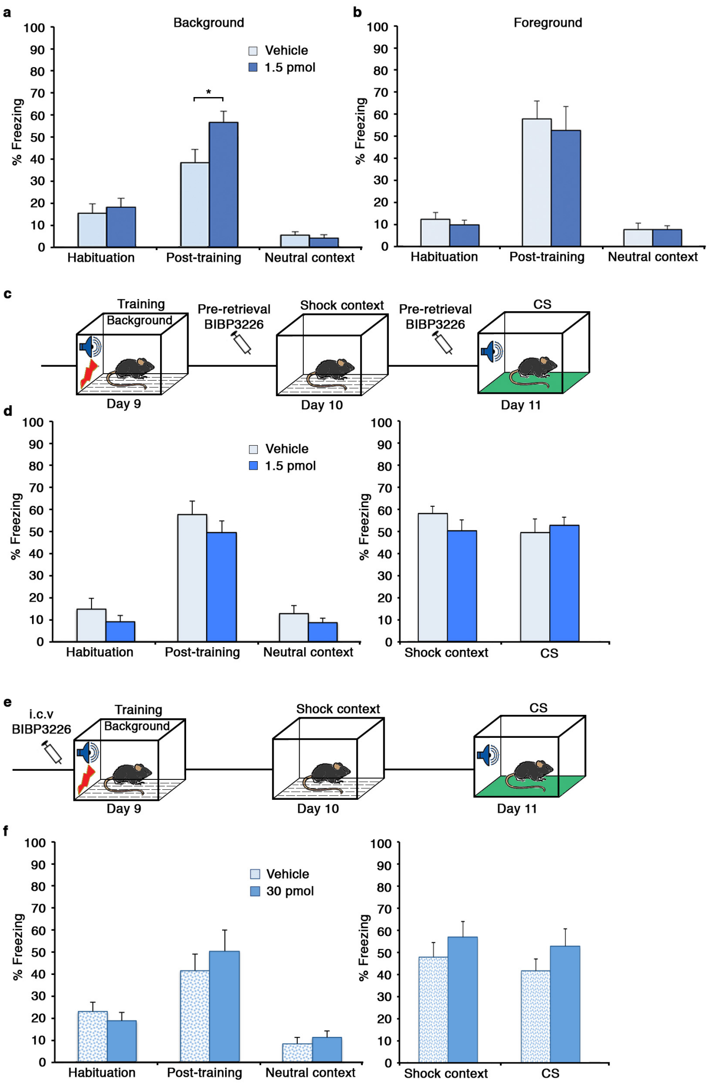

Supplementary Figure 5. Specific effects of NPY receptor blockage (**a**) Local pre-training injection of BIBP3226 into the dorsal DG increases post-training freezing behavior, but not during habituation or in the neutral context. This indicates increased immediate contextual fear memory formation[67]. (**b**) No effects of BIB3226 on these parameters are seen in animals that received injections before foreground context conditioning. (**c**) A schematic of retrieval testing under NPY receptor blockage. (**d**) (Left) No difference between groups (*n*=8 BIBP3226; *n*=8 controls) was observed during pre-training habituation, immediate post-training or in neutral context retrieval. (Right) Neither background context nor cued fear memory was affected by the pre-

retrieval injections of BIBP3226. (**e**) A schematic of behavior testing under NPY receptor blockage with intracerebroventricular (i.c.v.) injection. (**f**) (Left) The performance during pre-training habituation and immediate post-training remains unaffected. (Right) No change is observed in background context or auditory fear memory of C57BL/6 mice compared to vehicle (n=8) following pre-training i.c.v. injection of BIBP3226 at the anxiogenic dose[61] of 30 pmol BIBP3226 (n=7). Data are means + s.e.m. Statistical analysis was done with Student's unpaired t-test. *$P < 0.05$.

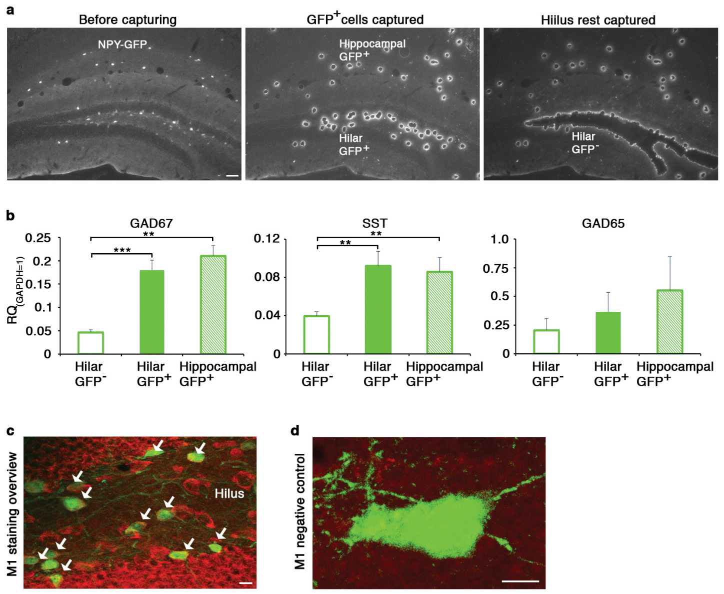

Supplementary Figure 6. HIPP cells and the M1 receptor (**a**) Microscopic images depict the dorsal hippocampus of an NPY-GFP mouse before and after laser microdissection. (Left) GFP^+ interneurons before isolation, (center) the same section after GFP^+ cells were collected from the hilus and cornu ammonis and (right) after collection of the remaining hilus. Scale bar, 100 µm. (**b**) High mRNA levels of GAD67 and SST are evident in GFP^+ cells of the hilus and CA1, compared to the GFP-negative tissue. This confirms the expected neurochemical signature of HIPP cells. GAD65 mRNA shows no difference. (**c**) M1 receptors (red) can be detected on the majority of GFP^+ cells (green) in the hilus of NPY-GFP mice, in addition to an expression on GFP^- cells in the granule cell layer. Scale bar, 10 µm. (**d**) A GFP^+ cell in the dorsal hilus of NPY-GFP mice, stained under omission of the M1 primary antibody to confirm the specificity of the M1 receptor immunolabeling shown in Fig. 4c. No labeling is evident on this cell body. Scale bar, 5 µm. Data are means + s.e.m. Statistical analysis was done with Fisher's LSD following one-way ANOVA. $^{**}P < 0.01$; $^{***}P < 0.001$.

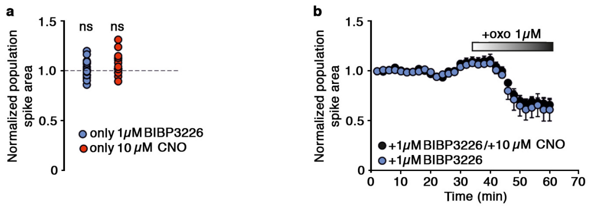

Supplementary Figure 7. Specificity of Y1 receptor blockage under muscarinic stimulation (a) Neither Y1 receptor blockage nor pharmacogenetic inactivation of HIPP cells alter the DG population spike area without pharmacological activation of muscarinic receptors (1 μM BIBP3226: *n*=16 slices and CNO: *n*=16 slices). **(b)** No difference is evident in the oxotremorine M induced depression of population spike responses following treatment with BIBP3226 (*n*=6 slices) or combined BIBP3226 and CNO (*n*=6 slices) in hM4Di transduced SST-CRE^{ERT2} driver mice. This indicates a major role of NPY transmission in the HIPP cell-mediated effect. Values are means ± s.e.m. Statistical analysis was done with paired *t*-test (**a**), Student's unpaired *t*-test **(b)**.

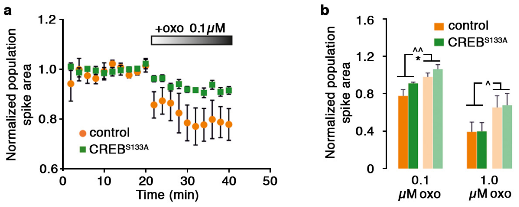

Supplementary Figure 8. CREB dependence of oxotremorine M effects (a) DG population spike development under low concentrations of oxotremorine M (oxo, 0.1 μM) and without BIBP3226 in mice expressing $CREB^{S133A}$ (*n*=9 slices) in comparison to controls (*n*=8 slices). **(b)** Summary graph comparing the average population spike response in mice expressing $CREB^{S133A}$ or control virus in the presence (*n*=7 $CREB^{S133A}$, *n*=5 control) or absence of BIBP3226. The depression of the DG population spike responses induced by a low-concentration of oxotremorine M (oxo, 0.1 μM) is reduced in mice expressing $CREB^{S133A}$. This effect is not seen at higher concentration (1 μM) of oxo. BIBP3226 attenuates the effect of oxo at both concentrations. Values are means ± s.e.m. Statistical analysis was done with two-way ANOVA. $CREB^{S133A}$ effect: *$P < 0.05$; BIBP3226 treatment effect: ^$P < 0.05$; ^^$P < 0.01$.

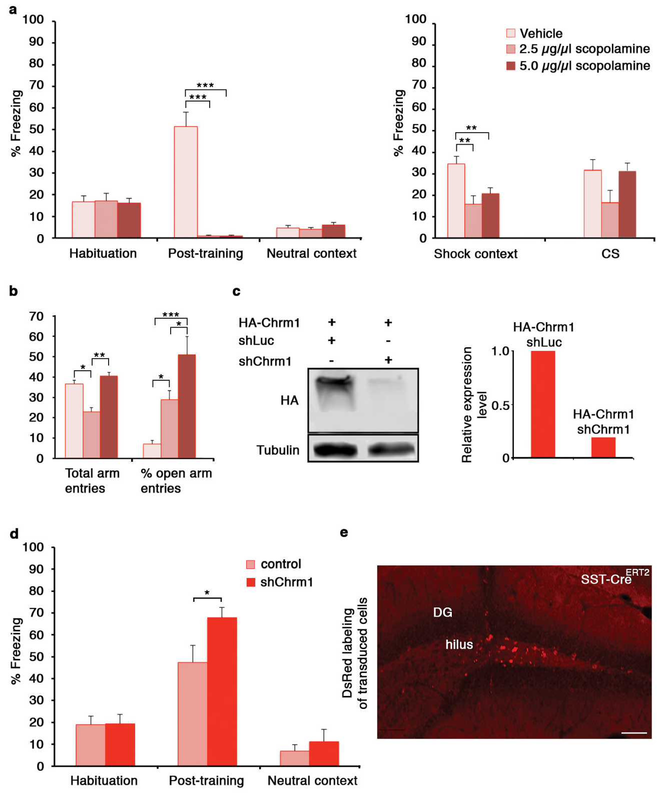

Supplementary Figure 9. Effects of pharmacological M1 receptor blockage in the DG and specificity of the Chrm1 knock down (**a**) Bilateral infusion of scopolamine before fear conditioning profoundly affects freezing during the immediate post-training test period, reducing it at both doses of 2.5 μg μl^{-1} (n=6) and 5.0 μg μl^{-1} (n=11) when compared to vehicle injected controls (n=10). Scopolamine furthermore reduces background context memory but does not significantly alter auditory cued memory. (**b**) Scopolamine injections (2.5 μg μl^{-1} n= 7, 5.0 μg μl^{-1} n= 10) to the dorsal DG induce an increased proportion of open arm entries in elevated plus maze, compared to vehicle controls (n=9), suggesting an anxiolytic-like effect. Thus pharmacological intervention with scopolamine does not specifically address the role of DG muscarinic transmission in contextual memory formation. (**c**) (Left) A western blot of proteins from NIH3T3 cells with heterologous expression of HA-tagged M1 receptors shows profound reduction of M1 expression after knockdown. Cells were co-transfected with HA-Chrm1 and either shLuc or shChrm1 as indicated. (Right)

Quantification of the M1 protein levels. (**d**) The knockdown of Chrm1 (n=8) in HIPP neurons increases the freezing response of mice during the immediate post-training period, indicating an effect on immediate contextual memory. The same effect is also observed upon blockage of NPY transmission in the DG, but not when HIPP neurons are silenced more generally with hM4Di receptors or with CREB^{S133A}. Habituation and neutral context responding are not affected compared to the control group expressing only the DsRed marker protein (n=7). (**e**) A representative microscopic image of viral DsRed expression in the hilus. Scale bar, 100 μm. Data are means + s.e.m. Statistical analysis was done with one-way ANOVA followed by Fisher's LSD in (**a, b**) and with Student's unpaired *t*-test in (**d**). *$P < 0.05$, **$P < 0.01$, ***$P < 0.001$.

Supplementary Table 1. Overview of statistical values

FIGURE NUMBER	TEST USED	ANNOVA	PAIRED COMPARISONS
1b	One-way ANOVA, post hoc Fisher's LSD	Shock context:<br>F(2.21) = 10.96, $P = 0.001$<br><br>CS:<br>F(2.21) = 16.77, $P < 0.001$	Shock context:<br>Background vs. foreground $P = 0.001$<br>Background vs. pure context $P < 0.001$<br>Foreground vs. pure context $P = 0.77$<br><br>CS:<br>Background vs. foreground $P = 0.006$<br>Background vs. pure context $P < 0.001$<br>Foreground vs. pure context $P = 0.12$
1e	Unpaired t-test		Background:<br>Shock context: t(16) = -2.50, $P = 0.023$<br>CS: t(16) = 1.18, $P = 0.25$<br><br>Foreground:<br>Shock context: t(13) = 0.45, $P = 0.66$<br>CS: t(13) = -1.21, $P = 0.24$
1h	Unpaired t-test		t(8) = -2.61, $P = 0.03$
2c	Unpaired t-test		Shock context: t(16) = -2.595, $P = 0.02$<br>CS: t(16) = 0.32, $P = 0.75$
2d	One-way ANOVA, post hoc Fisher's LSD	NPY:<br>F(2.16) = 30.221, $P = 0.001$<br><br>SST:<br>F(2.15) = 4.507, $P = 0.033$<br><br>GAD67:<br>F(2.16) = 18.595, $P = 0.001$	NPY:<br>Control vs. CREB: $P < 0.001$<br>Control vs. $CREB^{S133A}$: $P = 0.005$<br>CREB vs. $CREB^{S133A}$: $P < 0.001$<br><br>SST:<br>Control vs. CREB: $P = 0.059$<br>Control vs. $CREB^{S133A}$: $P = 0.013$<br>CREB vs. $CREB^{S133A}$: $P = 0.32$<br><br>GAD67 :<br>Control vs. CREB: $P < 0.001$<br>Control vs. $CREB^{S133A}$: $P = 0.74$<br>CREB vs. $CREB^{S133A}$: $P < 0.001$
2g	One-way ANOVA, post hoc Fisher's LSD	F(2.17) = 9.55, $P = 0.002$	Background vs. foreground: $P = 0.01$<br>Background vs. naïve: $P = 0.001$<br>Foreground vs. naïve: $P = 0.12$
3b	Unpaired t-test		Background<br>Shock context: t(18) = -3.40, $P = 0.003$<br>CS: t(18) = 0.98, $P = 0.364$<br><br>Foreground<br>Shock context: t(13) = 0.27, $P = 0.78$<br>CS: CS: t(13) = 1.10, $P = 0.28$
4a	One-way ANOVA, post hoc Fisher's LSD	NPY:<br>F(2.18) = 12.362, $P < 0.001$<br><br>Chrm1:<br>F(2.19) = 3.677, $P = 0.045$<br><br>Chrm2:<br>F(2.19) = 9.940, $P = 0.001$	NPY:<br>Hilar GFP^+ vs. Hilar GFP^-: $P < 0.001$<br>Hilar GFP^+ vs. Hippoc. GFP^+: $P = 0.615$<br>Hilar GFP^- vs. Hippoc. GFP^+: $P < 0.001$<br>Chmr1:<br>Hilar GFP^+ vs. Hilar GFP^-: $P =0.015$<br>Hilar GFP^+ vs. Hippoc. GFP^+: $P = 0.083$<br>Hilar GFP^- vs. Hippoc. GFP^+: $P = 0.451$<br><br>Chmr2:<br>Hilar GFP^+ vs. Hilar GFP^-: $P = 0.168$<br>Hilar GFP^+ vs. Hippoc. GFP^+: $P < 0.001$<br>Hilar GFP^- vs. Hippoc. GFP^+: $P = 0.008$
4d	Wilcoxon signed rank test		Resting membrane potential:<br>Control vs. oxo: $P = 0.0156$<br>Pirenzepine vs. oxo: $P = 0.1250$

			Input resistance:<br>Control vs. oxo: *P* = 0.0156<br>Pirenzepine vs. oxo: *P* = 0.1250<br><br>Spike frequency:<br>Control vs oxo: *P* = 0.0156<br>Pirenzepine vs. oxo: *P* = 1.0000
4e	Unpaired t-test		Oxo vs. BIBP3226+oxo: t(10) = -3.460. *P* = 0.006<br>Oxo vs. CNO+oxo: t(16) = -2.180, *P* = 0.045<br><br>(not shown in the figure)<br>Cch vs. BIB3226+Cch: t(16) = -2.353; *P* = 0.03<br>Cch vs. CNO+Cch: t(14) = -3.296; *P* = 0.005
5e	Unpaired t-test		Shock context: t(13) = -2.26, *P* = 0.04<br>CS: t(13) = -0.48, *P* = 0.53
6c,d	One-way repeated measures ANOVA, post hoc Fisher's LSD	F(2.14) = 28.701, *P* <0.001	CNO vs. aCSF: *P* = 0.006;<br>BIBP3226 vs. aCSF: *P* = 0.001<br>CNO vs. BIBP3226: *P* = 0.001
6e	Paired t-test		t (15) = 0.992, *P* = 0.337
6f	Unpaired t-test		t (22) = 2.340, *P* = 0.029
S1a	One-way ANOVA, post hoc Fisher's LSD	Habituation:<br>F(2.21) = 0.24, *P* = 0.78<br><br>Posttraining:<br>F(2.21) = 1.56, *P* = 0.23<br><br>Neutral context:<br>F(2.21) = 3.82, *P* = 0.03	Habituation:<br>N/A<br><br>Posttraining:<br>N/A<br><br>Neutral context:<br>Background vs. foreground *P* = 0.01<br>Background vs. pure context *P* = 0.09<br>Foreground vs. pure context *P* = 0.33
S1b	Unpaired t-test		Habituation: t(16) = -0.73, *P* = 0.47<br>Posttraining: t(16) = -0.52, *P* = 0.60<br>Neutral context: t(16) = 0.34, *P* = 0.73
S1c	Unpaired t-test		Habituation: t(13) = -0.35, *P* = 0.72<br>Posttraining: t(13) = -0.51, *P* = 0.91<br>Neutral context: t(13) = -0.22, *P* = 0.82
S1e	Unpaired t-test		Habituation: t(19) = 0.011, *P* = 0.991<br>Posttraining: t(19) = 0.261, *P* = 0.797<br>Neutral context: t(19) = 0.073, *P* = 0.942<br>Shock context: t(19) = 0.634, *P* = 0.534<br>CS: t(19) = 0.66, *P* = 0.53
S2b	Unpaired t-test		Habituation: t(13) = 1.01, *P* = 0.3<br>Posttraining: t(13) = 0.16, *P* = 0.87<br>Neutral context: t(13) = 1.13, *P* = 0.27<br>Shock context: t(13) = -0.56, *P* = 0.58<br>CS: t(13) = 1.58, *P* = 0.13
S2d	Unpaired t-test		Habituation: t(18) = 0.85, *P* = 0.40<br>Posttraining: t(18) = 0.59, *P* = 0.55<br>Neutral context: t(18) = 0.69, *P* = 0.49<br>Shock context: t(18) = -1.14, *P* = 0.26<br>CS: t(18) = 1.07, *P* = 0.32
S3b	Unpaired t-test		Habituation: t(14) = -0.66, *P* = 0.51<br>Posttraining: t(14) = -0.05, *P* = 0.95<br>Neutral context: t(14) = -0.50, *P* = 0.62<br>Shock context: t(14) = 2.25, *P* = 0.04<br>CS: t(14) = 0.53, *P* = 0.60
S4a	Unpaired t-test		Habituation: t(16) = 1.696, *P* = 0.109<br>Posttraining: t(16) = -2.09, *P* = 0.053<br>Neutral context: t(16) = -0.326, *P* = 0.749
S4b	Unpaired t-test		Distance: t(16) = -1.862, *P* = 0.081

			%Center time: t(16) = 0.444, *P* = 0.66
S4e	One-way ANOVA	F(2.17) = 0.12, *P* = 0.89	N/A
S5a	Unpaired t-test		Habituation: t(18) = -0.45, *P* = 0.65<br>Posttraining: t(18) = -2.59, *P* = 0.01<br>Neutral context: t(18) = 0.59, *P* = 0.56
S5b	Unpaired t-test		Habituation: t(13) = 0.67, *P* = 0.51<br>Posttraining: t(13) = 0.38, *P* = 0.70<br>Neutral context: t(13) = 0.01, *P* = 0.98
S5d	Unpaired t-test		Habituation: t(14) = 1.02, *P* = 0.32<br>Posttraining: t(14) = 0.96, *P* = 0.35<br>neutral context: t(14) = 0.93, *P* = 0.36<br>Shock context: t(14) =1.37, *P* = 0.19<br>CS: t(14) = 0.62, *P* = 0.54
S5f	Unpaired t-test		Habituation: t(13) = 0.71, *P* = 0.48<br>Posttraining: t(13) = -0.76, *P* = 0.46<br>Neutral context: t(13) = -0.62, *P* = 0.54<br>Shock context: t(13) = -0.94, *P* = 0.36<br>CS: t(13) = -1.16, *P* = 0.28
S6b	One-way ANOVA, post hoc Fisher's LSD	SST:<br>F(2.21) = 6.46, *P* = 0.007<br><br>GAD67:<br>F(2.18) = 25.094, *P* < 0.001<br><br>GAD65:<br>F(2.15)1.206, *P* = 0.32	SST:<br>Hilar GFP^+ vs. Hilar GFP^- *P* = 0.004<br>Hilar GFP^+ vs. Hippoc. GFP^+: *P* = 0.84<br>Hilar GFP^- vs. Hippoc. GFP^+: *P* = 0.007<br><br>GAD67:<br>Hilar GFP^+ vs. Hilar GFP^-: *P* < 0.001<br>Hilar GFP^+ vs. Hippoc. GFP^+: *P* = 0.30<br>Hilar GFP^- vs. Hippoc. GFP^+: *P* = 0.001<br><br>GAD65:<br>N/A
S7a	Paired t-test		BIBP3226 vs. aCSF: t(15) = 0.992, *P* = 0.337<br>CNO vs. aCSF: t(15) = 2.031, *P* = 0.06
S7b	Paired t-test		BIBP3226 vs. BIBP3226+CNO:<br>t(10) = -0.490, *P* =0.635
S8b	Two-way ANOVA	Low $CREB^{S133A}$:<br>F(1.25) = 5.013, *P* = 0.034;<br><br>BIBP3226:<br>F(1.25) = 13.543, *P* = 0.001<br><br>Interaction:<br>F(1.25) = 0.291, *P* = 0.594<br><br>High $CREB^{S133A}$:<br>F(1.25) = 0.016; *P* = 0.9<br><br>BIBP3226:<br>F(1.25) = 5.711, *P* = 0.025<br><br>Interaction:<br>F(1.25) = 0.006, *P* = 0.939	N/A
S9a	One-way ANOVA, post hoc Fisher's LSD	Habituation:<br>F(2.24) = 0.030; *P* = 0.970<br><br>Posttraining:<br>F(2.24) = 49.462; *P* < 0.001<br><br>Neutral context:<br>F(2.24) = 0.756; *P* = 0.480<br><br>Shock context:	Habituation<br>N/A<br><br>Posttraining:<br>2.5 $\mu g\ \mu l^{-1}$ scopolamine vs. vehicle: *P* < 0.001<br>5.0 $\mu g\ \mu l^{-1}$ scopolamine vs. vehicle: *P* < 0.001<br>2.5 $\mu g\ \mu l^{-1}$ vs. 5.0 $\mu g\ \mu l^{-1}$ scopolamine: *P* = 0.998<br><br>Neutral context<br>N/A<br><br>Shock context:

		F(2.24) = 7.861; *P* = 0.002<br><br>CS:<br>F(2.24) = 2.50, *P* = 0.1	2.5 μg $μl^{-1}$ scopolamine vs. vehicle: *P* = 0.002<br>5.0 μg $μl^{-1}$ scopolamine vs. vehicle: *P* = 0.005<br>2.5 μg $μl^{-1}$ vs. 5.0 μg $μl^{-1}$ scopolamine: *P* =0.357<br><br>CS:<br>N/A
S9b	Kruskal-Wallis<br><br>One-way ANOVA, post hoc Fisher's LSD	Total arm entries:<br>H(2) = 13.530; *P* = 0.001<br><br>%open arm entries;<br>F(2.23) = 13.14, *P* < 0.001	Total arm entries.<br>2.5 μg $μl^{-1}$ scopolamine vs. vehicle: *P* = 0.023<br>5.0 μg $μl^{-1}$ scopolamine vs. vehicle: *P* = 0.34<br>2.5 μg $μl^{-1}$ vs. 5.0 μg $μl^{-1}$ scopolamine: *P* = 0.001<br><br>% open arm entries<br>2.5 μg $μl^{-1}$ scopolamine vs. vehicle: *P* = 0.02<br>5.0 μg $μl^{-1}$ scopolamine vs. vehicle: *P* < 0.001<br>2.5 μg $μl^{-1}$ vs. 5.0 μg $μl^{-1}$ scopolamine: *P* = 0.02
S9d	Unpaired t-test		Habituation: t(13) = -0.076, *P* = 0.94<br>Posttraining: t(13) = -2.335, *P* = 0.03<br>Neutral context: t(13) = -0.664, *P* = 0.51

Supplementary Table 2. mRNA expression levels in GFP^+ interneurons of NPY-GFP mice of candidate factors not shown in Figure 4a or S5b. mRNA levels are expressed as reaction quotient relative to the housekeeping gene Glycerinaldehyd-3-phosphat-Dehydrogenase (GAPDH)

	Hilar GFP^+	Hilar GFP^-	Hippo-campal GFP^+	Statistics	Paired comparison of hilar GFP^+	
	RQ(GAPDH) Mean ± SEM	RQ(GAPDH) Mean ± SEM	RQ(GAPDH) Mean ± SEM	ANOVA for cell type	to hilar GFP^-	to hippo-campal GFP^+
CCK (Cholecystokinin)	0.0315 ± 0.00833	0.0482 ± 0.0038	0.0482 ± 0.00 619	F (2.21) = 2.294; *P* = 0.126	no difference	no difference
Chrm3 (Cholinergic receptor, M3)	0.0069 ± 0.00331	0.0172 ± 0.00315	0.0251 ± 0.00642	F (2.18) = 4.017; *P* = 0.036	no difference	*P* = 0.011
Chrm4 (Cholinergic receptor ,M4)	0.8653 ± 0.00807	0.8807 ± 0.00663	0.8587 ± 0.00731	F (2.20) = 2.329; *P* = 0.123	no difference	no difference
Grik1 (Glutamate receptor, ionotropic, kainate 1)	Not detected	Not detected	Not detected			
Grik2 (Glutamate receptor, ionotropic, kainate 2)	0.25 ± 0.09128	0.0834 ± 0.0164	0.0528 ± 0.00971	F (2.21) = 3.886; *P* = 0.037	*P* = 0.040	*P* = 0.017
Adra1d (Alpha 1 adrenergic receptor)	0.0452 ± 0.171	0.0513 ± 0.00479	0.0448 ± 0.00623	F (2.21) = 0.113; *P* = 0.894	no difference	no difference
5Ht2c (5 hydroxytrytamine (serotonin) 2C)	Not detected	Not detected	Not detected			
Drd2 (Dopamine receptor D2)	0.001 ± 0.00086	0.0199 ± 0.00171	0.0029 ± 0.00216	F (2.18) = 32.115; *P* < 0.001	*P* < 0.001	no difference
Drd3 (Dopamine receptor D3)	Not detected	Not detected	Not detected			
